# Cerebrospinal fluid lipidomic fingerprint of obstructive sleep apnoea in Alzheimer’s disease

**DOI:** 10.1186/s13195-023-01278-7

**Published:** 2023-08-07

**Authors:** Farida Dakterzada, Iván D. Benítez, Adriano Targa, Anna Carnes, Montse Pujol, Mariona Jové, Olga Mínguez, Rafi Vaca, Manuel Sánchez-de-la-Torre, Ferran Barbé, Reinald Pamplona, Gerard Piñol-Ripoll

**Affiliations:** 1 Unitat Trastorns Cognitius, Cognition and Behaviour Study Group, Santa Maria University Hospital, IRBLleida, Rovira Roure No. 44, Lleida, 25198 Spain; 2 Group of Translational Research in Respiratory Medicine, Hospital Universitari Arnau de Vilanova and Santa Maria, IRBLleida, Lleida, Spain; 3 Center for Biomedical Research in Respiratory Diseases Network (CIBERES), Madrid, Spain; 4 Department of Experimental Medicine, University of Lleida-Biomedical Research Institute of Lleida (UdL-IRBLleida), Lleida, Spain; 5 Department of Nursing and Physiotherapy, Group of Precision Medicine in Chronic Diseases, University Hospital Arnau de Vilanova and Santa María, IRBLleida, Faculty of Nursing and Physiotherapy, University of Lleida, Lleida, Spain

**Keywords:** Alzheimer’s disease, Biomarker, Cerebrospinal fluid, Diagnosis, Lipidomics, Obstructive sleep apnoea, STOP-Bang questionnaire

## Abstract

**Background:**

Obstructive sleep apnoea (OSA) has a high prevalence in patients with Alzheimer’s disease (AD). Both conditions have been shown to be associated with lipid dysregulation. However, the relationship between OSA severity and alterations in lipid metabolism in the brains of patients with AD has yet to be fully elucidated. In this context, we examined the cerebrospinal fluid (CSF) lipidome of patients with suspected OSA to identify potential diagnostic biomarkers and to provide insights into the pathophysiological mechanisms underlying the effect of OSA on AD.

**Methods:**

The study included 91 consecutive AD patients who underwent overnight polysomnography (PSG) to diagnose severe OSA (apnoea-hypopnea index ≥ 30/h). The next morning, CSF samples were collected and analysed by liquid chromatography coupled to mass spectrometry in an LC-ESI-QTOF-MS/MS platform.

**Results:**

The CSF levels of 11 lipid species were significantly different between AD patients with (*N* = 38) and without (*N* = 58) severe OSA. Five lipids (including oxidized triglyceride OxTG(57:2) and four unknown lipids) were significantly correlated with specific PSG measures of OSA severity related to sleep fragmentation and hypoxemia. Our analyses revealed a 4-lipid signature (including oxidized ceramide OxCer(40:6) and three unknown lipids) that provided an accuracy of 0.80 (95% CI: 0.71–0.89) in the detection of severe OSA. These lipids increased the discriminative power of the STOP-Bang questionnaire in terms of the area under the curve (AUC) from 0.61 (0.50–0.74) to 0.85 (0.71–0.93).

**Conclusions:**

Our results reveal a CSF lipidomic fingerprint that allows the identification of AD patients with severe OSA. Our findings suggest that an increase in central nervous system lipoxidation may be the principal mechanism underlying the association between OSA and AD.

**Supplementary Information:**

The online version contains supplementary material available at 10.1186/s13195-023-01278-7.

## Introduction

Alzheimer’s disease (AD) is the most common cause of dementia and is typically characterized by initial memory impairment, gradual affectation of other cognitive abilities, and ultimately disability and death [1]. The global burden of dementia was estimated at 57.4 million cases in 2019 and is expected to increase to 152.8 million cases in 2050 [2]. Currently, there is no cure available for AD. Therefore, identifying modifiable risk factors would help to reduce the incidence and slow the progression of this disease.

AD is characterized by the accumulation of extracellular abnormally folded amyloid-beta (Aβ) protein known as amyloid plaques, intracellular aggregations of hyperphosphorylated tau (Ptau) protein known as neurofibrillary tangles (NFTs), and neuronal loss in the brain [3]. The aetiology of AD is unknown, and it is believed that both genetic and environmental factors play a role in AD development [4]. Additionally, AD is normally accompanied by other comorbidities, such as diabetes, hypertension, depression, and sleep disorders [5].

Obstructive sleep apnoea (OSA) is the most common sleep-related breathing disorder and is characterized by recurrent episodes of complete or partial collapse of the upper airway during sleep. OSA has a high prevalence in patients with AD, and approximately 40% of AD patients have been reported to suffer from severe OSA [6]. The levels of Aβ42 and Ptau proteins were reported to be higher in the CSF of cognitively healthy elderly subjects with OSA [7] and subjects in the continuum of AD suffering from OSA [8]. In addition, longitudinal studies have suggested an increase in the speed of cerebral amyloid accumulation promoted by OSA [9]. However, the results regarding the effect of OSA on the cognition of patients with mild cognitive impairment (MCI) and AD are not consistent [10–12]. Importantly, OSA and AD share many risk factors, including cardiovascular risk factors [5, 13]. Therefore, given the strong link between AD and OSA and the high prevalence of OSA in patients with AD, the diagnosis of this comorbidity is essential in the AD population.

Polysomnography (PSG) is the technique of choice for the detection and characterization of OSA. However, applying this method to AD patients is difficult because it requires sleeping for one or two nights in the hospital and as well as patient collaboration [14]. In addition, screening questionnaires, including the STOP-Bang questionnaire (SBQ) [15] and the Berlin questionnaire (BQ) [16], have been shown to be insufficient for identifying AD patients at risk of OSA [17]. Thus, searching for new screening tools to detect OSA among AD patients is of great importance.

OSA is strongly associated with lipid dyshomeostasis [18]. In addition, OSA can increase lipoxidation, a harmful condition that has been evidenced at both central and systemic levels in AD patients [19, 20]. As a result, the identification of alterations in lipid species could contribute to discovering OSA-related lipid profiles in AD and improve our knowledge of the relationship between these two complex pathological conditions. Lipidomics is the study of the large-scale detection, identification, and quantification of individual lipid species in biological samples and has been demonstrated to be a powerful tool for biomarker discovery. In this regard, cerebrospinal fluid (CSF) would be an interesting and reliable source for examination because it is in contact with the extracellular space of brain cells. In addition, AD biomarkers are measured in CSF, and therefore, this biological fluid can be used for both purposes.

Therefore, the aim of our study was (i) to investigate the differences in the levels of CSF lipids between AD patients with and without severe OSA, which would be of great relevance for reliable screening of OSA among AD patients; (ii) to evaluate whether the severity of OSA is correlated with alterations in CSF lipid levels; and (iii) to assess the diagnostic performance of CSF lipidomic findings in combination with classic screening tests such as the SBQ.

## Materials and methods

### Study population

This is an ancillary study from trial NCT02814045 that was conducted in the Cognitive Disorders Unit of the Hospital Universitari Santa Maria (Lleida, Spain) from November 2014 to November 2017 to assess the cognitive evolution of AD patients with and without OSA after 1 year of follow-up. The patients were recruited prospectively and consecutively according to the following inclusion criteria: (1) males and females above 60 years with a new diagnosis of mild or moderate AD (Mini-Mental State Examination (MMSE) score ≥ 20) according to the National Institute on Ageing – Alzheimer’s Association (NIA-AA) criteria [21] and without specific treatment for dementia at the moment of inclusion; (2) lack of hearing or visual problems that, in the investigator’s judgement, would interfere with the compliance of the neuropsychological examination; (3) signed informed consent from the patient and the responsible caregiver (and/or if applicable, the legal representative if different from the responsible caregiver); and (4) existence of a reliable and knowledgeable caregiver accompanying the patient to all clinic visits during the study.

The exclusion criteria were as follows: (1) a previous diagnosis of OSA treated with continuous positive airway pressure; (2) patients with mild-moderate AD with current acetylcholinesterase inhibitor treatment or memantine, diagnosis of severe AD, or other types of dementia; (3) any previously diagnosed sleep disorder including narcolepsy, severe insomnia, or chronic lack of sleep; (4) comorbidities such as cancer, severe depression, severe renal or hepatic insufficiency, and severe cardiac or respiratory failure; and (5) excessive somnolence for unknown reasons. All exclusion criteria are available in the paperwork for NCT02814045.

### Study design

Patients included in the study underwent a detailed interview regarding anthropometric data, personal psychiatric history, and a general clinical examination for associated conditions and comorbidities. At baseline, participants were evaluated by a polysomnographic study, and blood and CSF samples were collected after PSG to determine the APOE genotype and the levels of Aβ42, total tau (Ttau), and Ptau. Based on the PSG findings, the study population was classified as severe OSA (apnoea-hypopnea index [AHI] ≥ 30/h) and nonsevere OSA (AHI < 30/h) patients.

### Clinical variables

The cognitive state of all participants was assessed using the MMSE [22]. A semistructured sleep questionnaire for detecting OSA, which included the SBQ, was administered to sixty-two patients. The SBQ is an 8-item questionnaire that assesses the presence or absence of snoring, tiredness, observed sleep apnoea, high blood pressure, body mass index (BMI), age, neck circumference, and sex. The score ranges from 0 to 8, and a score of ≥ 3 is considered to indicate a high risk of moderate/severe OSA [15]. Excessive daytime sleepiness was indicated by an Epworth Sleepiness Scale (ESS) score > 10 [23]. BMI was calculated as body weight (in kg)/height (in m^2^). The other collected variables included age, sex, years of education, alcohol consumption, smoking, vascular risk factors (hypertension, dyslipidaemia, stroke, diabetes mellitus, and heart diseases), and personal psychiatric history.

### Polysomnography (PSG)

To classify the patients as nonsevere OSA (AHI < 30/h) or severe OSA (AHI ≥ 30/h) patients, PSG was performed according to the international guidelines. The following devices were used: a Sibelmed Exea Series 5 (Sibel SAU, Spain), a Philips Respironics Alice 6 LDx (Philips, USA), an Embletta® sleep monitor (Embla, Canada), and an ApneaLink Resmed (Resmed, Canada).

Parameters related to OSA severity were defined as follows: Apnoea was defined as the absence of airflow for more than 10 s. Hypopnea was defined as a reduction in airflow that lasted more than 10 s leading to arousal or oxygen desaturation (represented by a decrease in oxygen saturation greater than 3%). The AHI was defined as the number of apnoea and hypopnoea events per hour during the time spent sleeping. CT90 was defined as the percentage of cumulative sleep time with oxygen saturation (SaO_2_) < 90%. The arousal index was defined as the number of awakening events per hour after sleep onset.

### Sample collection

Fasting blood and CSF samples were collected between 8:00 and 10:00 a.m. after PSG to avoid variations related to circadian rhythm. CSF samples were collected in polypropylene tubes by a lumbar puncture at levels L4/L5. The samples were centrifuged at 2000 × *g* for 10 min at 4 °C to exclude insoluble material. Blood samples were collected in EDTA-containing tubes. The buffy coat was separated by centrifugation of blood samples at 1500 rpm for 20 min. All samples were aliquoted and immediately stored at − 80 °C until use.

### APOE genotyping

DNA was extracted from buffy coat cells using a Maxwell® RCS Buffy Coat DNA kit (Promega, USA). APOE genotyping was performed using two TaqMan® SNP genotyping assays (C_3038793_20 and C_904973_10) and real-time polymerase chain reaction (PCR) according to the manufacturer’s user guide (Publication No. MAN0009593, revision B.0).

### Measurement of AD biomarkers

The levels of CSF Aβ42 (Innotest® β-amyloid (1–42)), Ttau (Innotest® hTAU Ag), and Ptau (Innotest® phospho-tau (181P)) were determined by the enzyme immunoassay method according to the manufacturer’s instructions (Fujirebio Europe, Ghent, Belgium). All samples were measured in duplicate and expressed in pg/mL. We considered Aβ42 values < 600 pg/mL, Ttau > 425 pg/mL, and Ptau > 65 pg/mL as positive/abnormal [24].

### Lipidomic profiling

An untargeted lipidomic platform was used to determine the CSF lipidome of patients. The lipids were extracted from 90 µL CSF samples using a methyl tert butyl ether-based validated method [25, 26]. Class representative internal standards (Additional file 1: Table S1) were added to the extraction solvent to check lipid species retention time, to evaluate lipid extraction for each sample, and to use as an internal standard for the semiquantitative approach used. Lipid extracts were analysed via ultrahigh-performance liquid chromatography (UHPLC) coupled with electrospray ionization quadrupole time of flight (ESI-Q-TOF) tandem mass spectrometry (MS/MS) according to a previously published method [27, 28]. The equipment was an Agilent 1290 liquid chromatography system (Agilent Technologies, Santa Clara, CA, USA) coupled with a 6520 ESI-Q-TOF mass spectrometer (Agilent Technologies, Santa Clara, CA, USA). The order for the injection of samples was randomized, and quality control (QC) samples were used to control instrumental drift. QC samples were pools of all the samples distributed in different aliquots and inserted in every five real samples. Data were acquired in both positive and negative ionization modes. For MS/MS confirmation, the same parameters used for MS analyses were applied, adding collision voltages of 0 V, 10 V, 20 V, and 40 V. Data were acquired using the MassHunter Data Acquisition software (Agilent Technologies, Barcelona, Spain) and preprocessed using the MassHunter Mass Profiler Professional software (Agilent Technologies, Barcelona, Spain), as previously described [29]. Only those features with a minimum of 2 ions were selected. Compounds from different samples were aligned using retention time windows of 0.1% ± 0.25 min and 30.0 ppm ± 2.0 mDa. Only stable features (found in at least 70% of the QC samples) were considered for the analysis and to correct individual bias [30]. The signal was corrected using a LOESS approach [31].

### Lipid identification

The potential identities of the differentially expressed features were searched in the Human Metabolome Database (HMDB) [32] according to the exact mass and retention time, while the molecular weight tolerance was adjusted to 30 ppm. Potential identities were confirmed by comparison of the exact mass, retention time, and MS/MS spectral fragmentation pattern of the class representative internal standards, when available, with the public database [29].

### Statistical analyses

Descriptive statistics were used to determine the characteristics of the study population. The normality of the distributions was assessed by the Shapiro–Wilk test. Normally distributed continuous data were summarized using the mean (standard deviation), and nonnormally distributed continuous data were summarized using the median (25th percentile; 75th percentile). Categorical data were summarized using frequency (percentage). Clinical and sociodemographic characteristics of the patients were compared between the severe OSA (AHI ≥ 30) and nonsevere OSA (AHI < 30) groups using Student’s *t* test (or an equivalent nonparametric test) or the chi-squared test for quantitative and categorical variables, respectively. Lipid levels were log-transformed for statistical purposes. Missing data were not imputed because there was not enrolled any missing variable in the differential expression analysis between the study groups. Linear models with empirical Bayes statistics were used to evaluate the differences in lipid levels between the groups [33] while controlling for age, sex, and BMI. Differential expression of lipid species was defined as a significant difference (*p* value) < 0.05 and a fold change (FC) > 1.25 (or < 0.8 for downregulated lipids) between the groups. Due to the exploratory nature of the study, *p* values were not adjusted for multiple comparisons.

A partial least squares-discriminant analysis (PLS-DA) was performed with differentially expressed lipids including the first two components. In addition, the variable’s importance in projection scores (VIP) of lipids on the first two components of the PLS-DA analysis was quantified. To evaluate nonlinear and complex interactions between differentially expressed lipids, the variable’s importance scores (VI), calculated as the average of 50 runs of random forests, were determined for each differentially expressed lipid. Dose–response relationship between the levels of differentially expressed lipids and PSG parameters was assessed using a linear model adjusted for age, sex, and BMI. To compare the magnitude of association, the lipids levels were standardized in this analysis.

To construct a lipidomic signature that predicted severe OSA, a selection model process was performed. First, a feature selection process based on random forest [34] was performed to construct a lipidomic signature that predicted severe OSA. This process is suitable for high-dimensional data. The feature selection process was applied on differentially expressed lipids and repeated 10 times to account for variability in the selection process. The lipids selected in some execution of the process were included in candidates set for the final predicted model. The candidates were included as predictors in a logistic model with OSA status as a response (severe or nonsevere OSA). The best model, based on the Akaike Information Criterion (AIC), included the lipids that composed the final lipidomic signature. Receiver operating characteristic (ROC) curves were constructed for the selected model. Additionally, we assessed the predictive ability of the selected model lipidomic fingerprint, the reference questionnaire (SBQ), and its combination using the area under the ROC curve (AUC) as the global discrimination value measure.

Furthermore, to determine the association of each lipid with the differential diagnosis, we performed a multivariate regression analysis using a backward selection procedure. The statistical significance was set at *p* values < 0.05. All statistical analyses were performed using the R software, version 4.0.2.

## Results

### Characteristics of the cohort

A total of 91 consecutive mild-moderate AD patients were included in the study. The median (p_25_; p_75_) age of the population was 76.0 (72.0; 80.0) years; 54 (59.3%) participants were women, and the median MMSE score was 23.0 (22.0; 25.0) points. Arterial hypertension was the most frequent vascular risk factor, as it was present in 52 (57.1%) patients, followed by dyslipidaemia in 43 (47.3%) participants and diabetes in 18 (19.8%) participants. Regarding the sleep parameters, the median ESS was 5.00 (3.00; 8.00), and the median AHI was 23.9 (13.7; 50.3). Fifty-three patients were considered to have nonsevere OSA (AHI < 30), and 38 patients were considered to have severe OSA (AHI ≥ 30). The baseline characteristics of the patients were similar between the severe OSA and nonsevere OSA groups. The baseline characteristics for the severe OSA and nonsevere OSA groups are summarized in Table 1.

**Table 1:** Characteristics of the study population according to the severity of obstructive sleep apnoea (OSA)

	**ALL (*N* = 91)**	**Nonsevere OSA (*N* = 53)**	**Severe OSA (*N* = 38)**	***p* value**
**Demographic data**				
Age (years), median [IQR]	76.0 [72.0; 80.0]	75.0 [72.0; 80.0]	78.5 [73.0; 80.0]	0.178
Gender (female), *n* (%)	54 (59.3%)	36 (67.9%)	18 (47.4%)	0.08
BMI (kg/m^2^), median [IQR]	27.7 [24.9; 30.9]	27.4 [24.6; 30.2]	28.1 [26.6; 32.7]	0.022
APOE4 (yes), *n* (%)	47 (51.6%)	26 (49.1%)	21 (55.3%)	0.71
Family history of AD (yes), *n* (%)	35 (38.5%)	17 (32.1%)	18 (47.4%)	0.208
**Comorbidities**				
Hypertension (yes), *n* (%)	52 (57.1%)	30 (56.6%)	22 (57.9%)	1
Diabetes mellitus (yes), *n* (%)	18 (19.8%)	12 (22.6%)	6 (15.8%)	0.588
Dyslipidemia (yes), *n* (%)	43 (47.3%)	25 (47.2%)	18 (47.4%)	1
Stroke (yes), *n* (%)	5 (5.49%)	2 (3.77%)	3 (7.89%)	0.646
Depression (yes), *n* (%)	28 (30.8%)	17 (32.1%)	11 (28.9%)	0.929
Smoker				0.763
Nonsmoker, *n* (%)	73 (80.2%)	41 (77.4%)	32 (84.2%)	
Current smoker, *n* (%)	1 (1.10%)	1 (1.89%)	0 (0.00%)	
Former smoker, *n* (%)	17 (18.7%)	11 (20.8%)	6 (15.8%)	
**AD CSF biomarkers**				
Aβ42 (pg/mL), median [IQR]	493 [399; 580]	489 [393; 584]	505 [406; 564]	0.679
Total tau (pg/mL), median [IQR]	494 [350; 696]	494 [369; 707]	469 [346; 684]	0.676
Phosphorylated tau (pg/mL), median [IQR]	81.0 [55.4; 97.5]	80.0 [58.0; 95.0]	81.0 [55.1; 98.0]	0.929
**Blood lipid levels**				
Total cholesterol (mg/dL), mean (SD)	206 (37.4)	208 (38.5)	204 (36.2)	0.613
LDL cholesterol (mg/dL), mean (SD)	123 (37.5)	121 (40.5)	126 (33.1)	0.559
HDL cholesterol (mg/dL), median [IQR]	57.5 [49.0; 65.0]	58.0 [48.2; 64.2]	56.0 [50.5; 65.0]	0.584
Triglyceride (mg/dL), median [IQR]	113 [88.0; 143]	118 [91.5; 142]	113 [82.0; 143]	0.557
**Polysomnography data**				
AHI	23.9 [13.7; 50.3]	15.1 [8.05; 19.5]	53.3 [42.8; 62.2]	< 0.001
Obstructive apnoea index	5.23 [0.61; 14.1]	1.22 [0.19; 5.17]	14.6 [7.68; 23.5]	< 0.001
Hypopnea index, median [IQR]	14.0 [7.86; 26.2]	10.1 [6.10; 13.9]	30.6 [22.2; 37.5]	< 0.001
Central apnoea index, median [IQR]	0.19 [0.00; 0.96]	0.00 [0.00; 0.45]	0.60 [0.00; 4.06]	0.007
Mixed apnoea index, median [IQR]	0.00 [0.00; 0.53]	0.00 [0.00; 0.19]	0.48 [0.00; 2.44]	< 0.001
Arousal index (event/h), mean (SD)	38.8 (17.4)	32.9 (16.2)	47.4 (15.5)	< 0.001
CT90, %	2.67 [0.40; 9.41]	1.18 [0.20; 3.79]	6.27 [1.48; 15.1]	0.002
Mean SaO_2_, %	93.0 [92.0; 94.0]	93.0 [92.0; 94.0]	92.8 [92.0; 93.0]	0.189
Minimum SaO_2_, %	84.0 [79.0; 87.0]	86.0 [82.0; 88.0]	81.0 [78.0; 85.0]	0.001
**Epworth Sleepiness Scale (0–24), median [IQR]**	5.00 [3.00; 8.00]	5.00 [2.00; 8.00]	5.50 [3.00; 8.00]	0.5
**MMSE**	23.0 [22.0; 25.0]	23.0 [22.0; 25.0]	24.0 [22.0; 25.0]	0.475
**Medications**				
Acetylcholinesterase inhibitors or memantine, *n* (%)	86 (94.5%)	50 (94.3%)	36 (94.7%)	0.999
ACE inhibitors, *n* (%)	25 (27.5%)	14 (26.4%)	11 (28.9%)	1
Beta-blockers, *n* (%)	14 (15.4%)	6 (11.3%)	8 (21.1%)	0.33
Diuretic agents, *n* (%)	24 (26.4%)	16 (30.2%)	8 (21.1%)	0.463
Calcium channel blockers, *n* (%)	12 (13.2%)	8 (15.1%)	4 (10.5%)	0.748
Lipid-lowering agents, *n* (%)	41 (45.0%)	22 (41.5%)	19 (50.0%)	0.522
Insulin, *n* (%)	2 (2.20%)	1 (1.89%)	1 (2.63%)	1

*BMI* body mass index, *AD* Alzheimer’s disease, *AHI* apnoea-hypopnea index per hour, *CSF* cerebrospinal fluid, *APOE Ɛ4* apolipoprotein E epsilon 4 allele, *MMSE* Mini-Mental State Examination, *ACE* angiotensin-converting enzyme, *OSA* obstructive sleep apnoea, *SaO*_*2*_ = oxygen saturation, *CT90* = time with SaO_2_ < 90%

### Quality control

After applying quality control on untargeted lipidomic determination, 201 lipids were detected and included in the analyses. A PCA analysis with a whole lipid profile did not show patients with significant distances to their corresponding centroid (Fig. S1).

### Differentially expressed lipids between AD patients with and without severe OSA

**Fig. 1:**
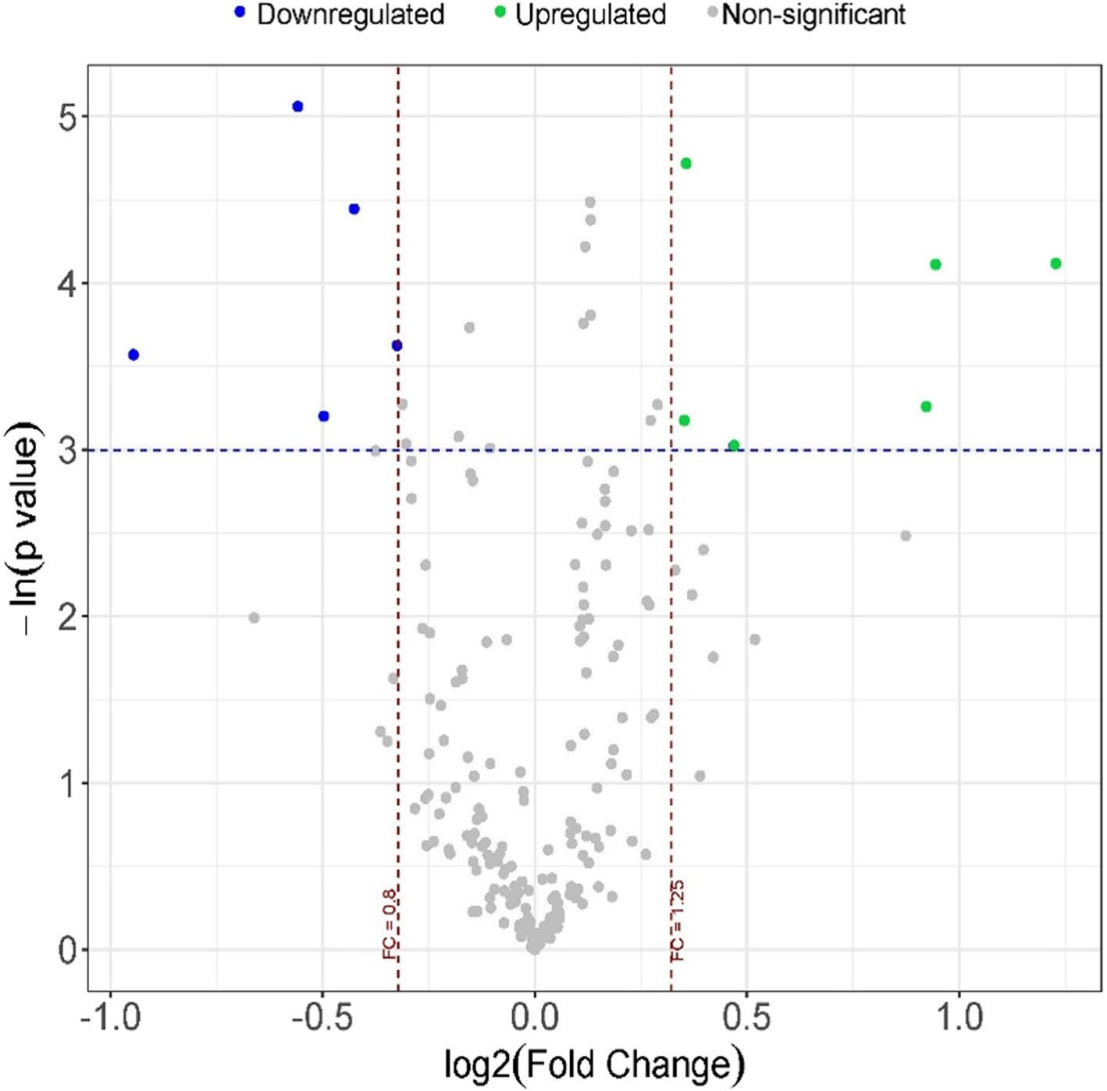
Lipidomic analysis in AD patients with severe OSA. Volcano plots of the FC (*x*-axis) and *p* value (*y*-axis) for each detected lipid species in the comparison of AD patients with and without severe OSA. Blue dots represent significantly downregulated (FC < 0.80) and green dots represent significantly upregulated (FC > 1.25) lipids in patients with severe OSA. The results were adjusted for age, sex, and BMI. The *p* value threshold defining statistical significance was < 0.05

**Fig. 2:**
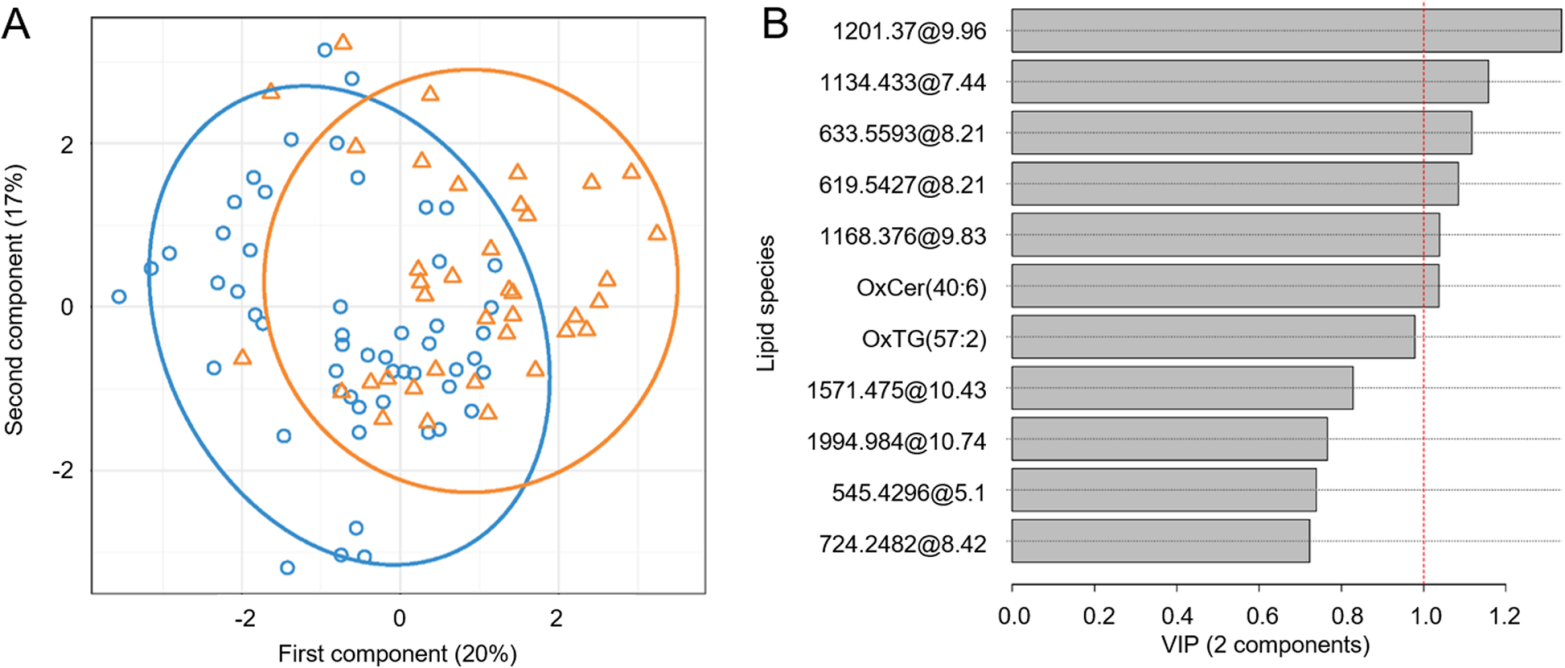
Partial least squares-discriminant analysis for differentially expressed lipids between patients with and without severe OSA. **A** Individual loading for the two first components. **B** The rank of variable importance of differentially expressed lipids on the two first components. Lipid features included in the final model. Feature selection was based on a combination of random forest and automated model selection using the Akaike Information Criterion. **B** Receiver operating characteristic curves for predicting severe OSA using the lipid signature. The AUC (95% CI) for the model is shown. OxCer, oxidized ceramide; OxTG, oxidized triglyceride; mass at RT, mass at retention time; OR, odds ratio

We evaluated the differences in the levels of lipids between patients with and without severe OSA. Following adjustment for confounding factors (age, sex, and BMI), 11 differentially expressed (DE) lipid species were identified, five with reduced (FC from 0.52 to 0.79) and six with increased CSF levels in patients with severe OSA (FC from 1.27 to 2.34) (Fig. 1 and Fig. S2). The PLS-DA analysis with DE lipids showed a lipid profile specific to each group (Fig. 2A). In this analysis, six DE lipids showed an VIP score higher than 1 (Fig. 2B). To evaluate nonlinear and complex interactions between differentially expressed lipids, we ranked the VI by conducting a random forest process (Fig. S3). On the other hand, five DE lipid species demonstrated a dose–response relationship, after adjustment for age, sex, and BMI, with some parameters of the PSG related to sleep fragmentation (arousal index, AHI, several respiratory events during sleep, and different measures of hypoxemia (average and minimum arterial oxygen saturation (SaO_2_), and the percentage of time with SaO_2_ < 90% (CT90)) (Additional file 1: Fig. S4 and Table S2).

### Identification of differentially expressed lipids

From 11 dysregulated lipid species between the diagnostic groups, two lipid species were identified. One was an oxidized ceramide (OxCer(40:6)), and the other was an oxidized triglyceride (OxTG(57:2)). The identity of the other nine differentially expressed lipid species remained unknown (Table 2).

**Table 2:** Differentially expressed lipids between AD patients with and without severe OSA

**Mass**	**RT (min)**	**Mass/z**	**Putative identity**	**Class**	**Fold change**	***p***
609.5135	8.21	608.5135	OxCer(40:6)	Sphingolipid	1.896	0.038
633.5593	8.21	632.5593	Unknown		2.343	0.016
619.5427	8.21	618.5427	Unknown		1.925	0.016
1168.376	9.83	1169.376	Unknown		0.799	0.027
1201.37	9.96	1202.37	Unknown		0.679	0.006
977.8707	10.24	978.8707	OxTG(57:2)	Glycerolipid	0.744	0.012
1994.984	10.74	1995.984	Unknown		1.281	0.009
1134.433	7.44	1135.433	Unknown		0.519	0.028
724.2482	8.42	725.2482	Unknown		0.708	0.041
545.4296	5.1	546.4296	Unknown		1.277	0.042
1571.475	10.43	1572.475	Unknown		1.384	0.049

*RT* retention time, *OxCer* oxidized ceramide, *OxTG* oxidized triglyceride

### Lipidomic prediction model for severe OSA

**Fig. 3:**
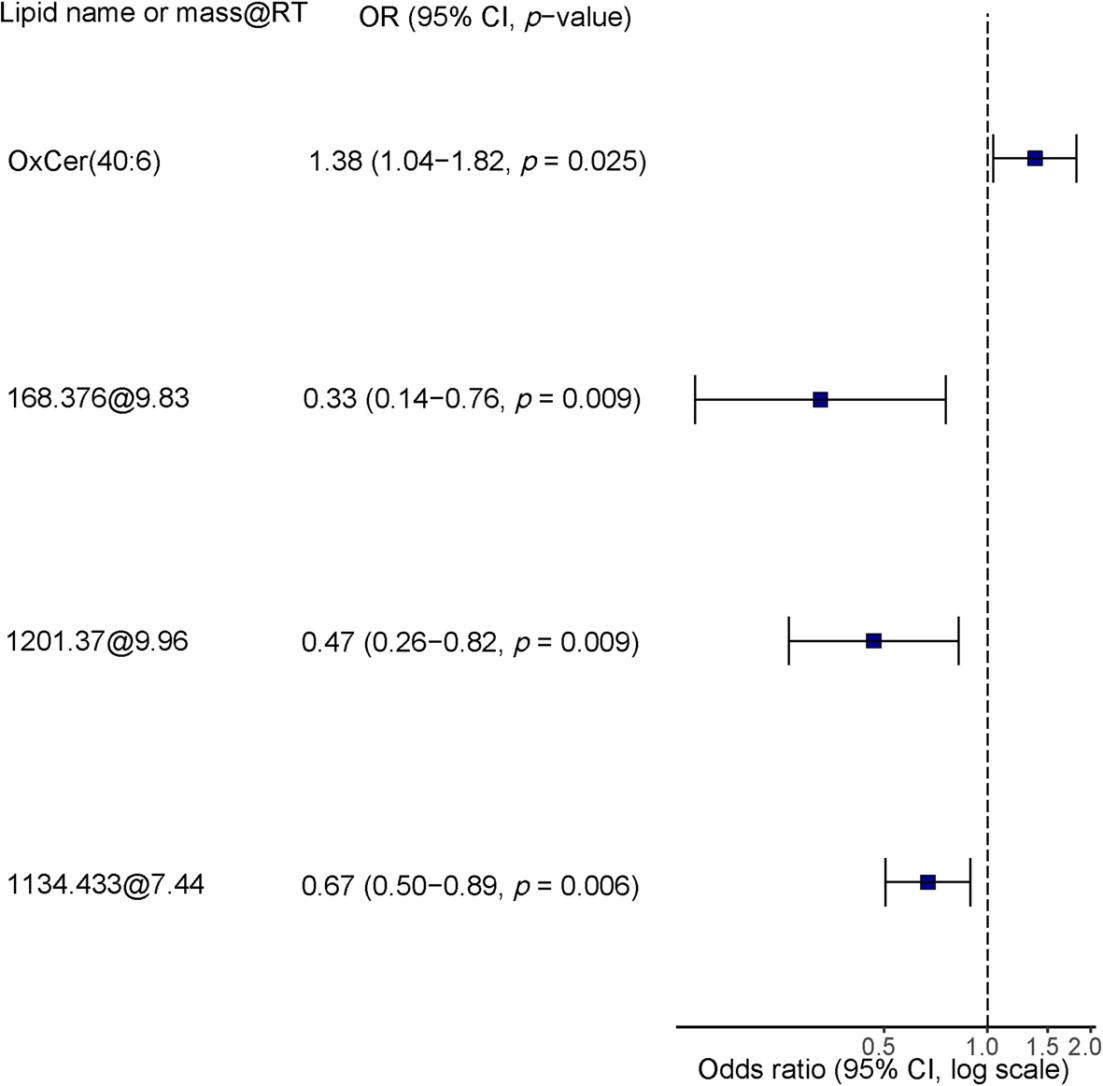
Potential CSF lipid signature for the diagnosis of severe OSA in AD patients. Lipid features included in the final model. Feature selection was based on a combination of random forest and automated model selection using the Akaike Information Criterion. OxCer, oxidized ceramide; mass at RT, mass at retention time; OR, odds ratio

**Fig. 4:**
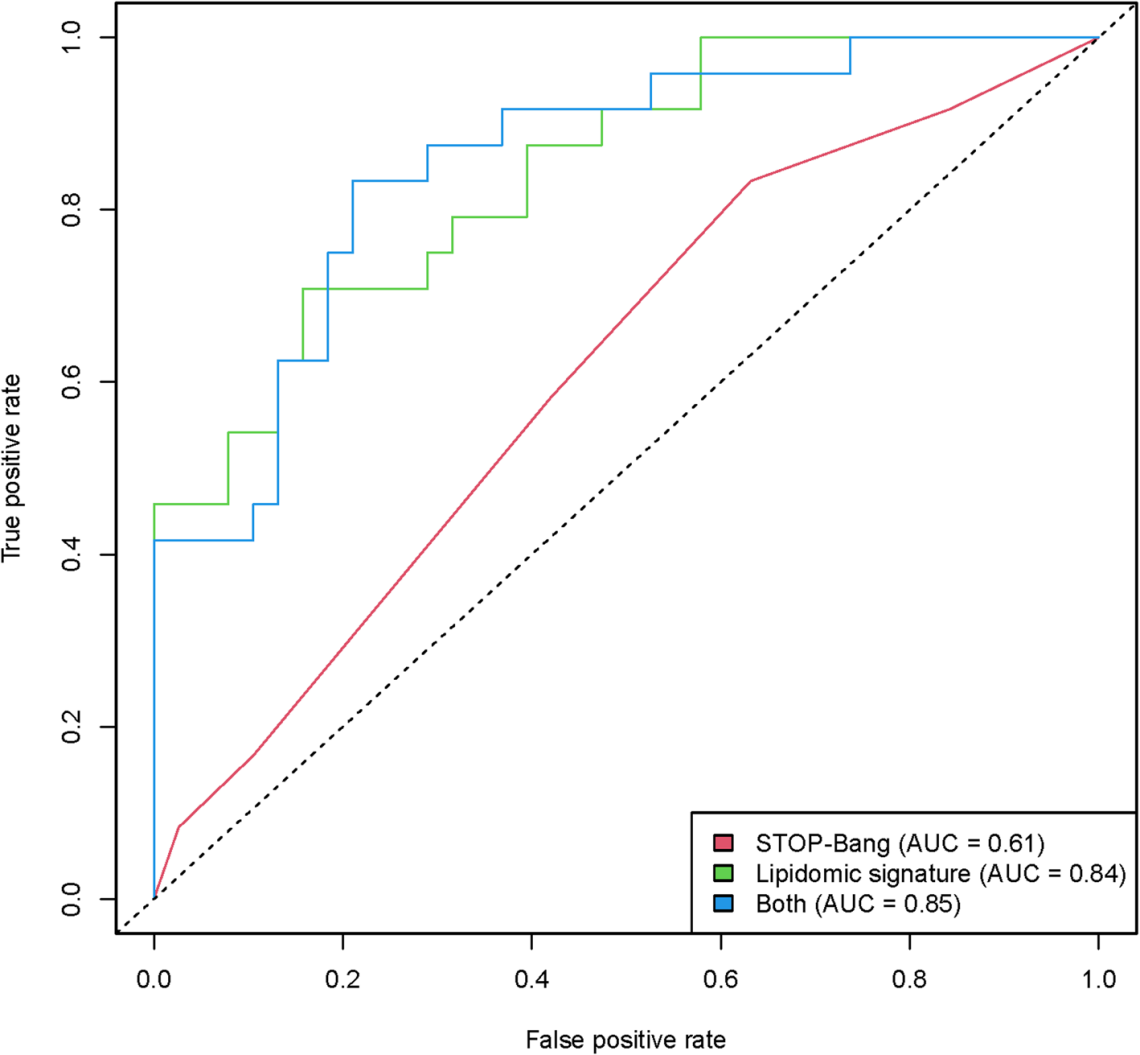
Receiver operating characteristic curves for predicting severe OSA in AD patients using the STOP-Bang questionnaire (red), lipid signature (green), and the combination of both methods (blue). Analysis was performed in those patients with STOP-Bang data. The AUC for each model is shown

One of the objectives of this study was to identify a CSF lipidomic signature that would allow the identification of AD patients with severe OSA. In the first step, the selection process of important DE lipids in the discrimination of severe OSA selected six lipids (OxCer(40:6), 619.5427@8.21, 1168.376@9.83, 1201.37@9.96, 545.4296@5.1, 1134.433@7.44). In the second step, the multivariate logistic models with combinations of the selected lipids are compared. Finally, the model selected, based on AIC, included a specific signature of severe OSA composed of four lipid species: OxCer(40:6) and three lipids with unknown identity (Fig. 3). The predictive performance of this lipid signature yielded an AUC of 0.80 (95% CI 0.71–0.89) in the detection of severe OSA in AD patients. Next, in a subpopulation with available SBQ (*N* = 62), we combined the SBQ and the lipid signature data. This combination increased the prediction power for severe OSA provided by the SBQ from AUC = 0.61 (95% CI 0.50–0.74) to AUC = 0.85 (95% CI 0.71–0.93) (Fig. 4). On the other hand, the multivariate regression analysis, using a backward selection procedure, included 7 lipids (Fig. S5).

## Discussion

To the best of our knowledge, this is the first study to investigate the OSA severity-associated lipid profile in the CSF of patients with AD. We detected 11 CSF lipids that were differentially expressed between AD patients with and without severe OSA, regardless of the incorporation of confounding factors. This lipidomic signature was significantly correlated with different polysomnographic measures related to OSA. In addition, the discriminating power of this lipid signature in separating AD patients with severe OSA from AD patients with nonsevere OSA was much greater than the use of the STOP-Bang screening questionnaire alone. Importantly, the identified lipids possibly indicate the harmful effects produced by severe OSA at the brain level.

AD is a neurodegenerative disease usually accompanied by several comorbidities, including hypertension, diabetes, dyslipidaemia, depression, and sleep disorders. These comorbid conditions have been shown to be related to an increased risk of AD and its progression [5]. OSA, which is the most common sleep disorder, has a high prevalence (45–90%) in patients with AD [6] and has been shown to be associated with increased AD pathology [8, 9] and disease incidence [35]. In addition, OSA is a risk factor for several AD-related comorbidities, and its underdiagnosis may be associated with greater difficulties in the control of blood pressure, increased insulin resistance, metabolic syndrome, or obesity, all factors that worsen the cognitive evolution of patients [36, 37]. The difficulties in performing PSG among patients with cognitive impairment, the high costs of the procedure, and the limited usefulness of the existing screening tests [17] in this population make it urgent to identify new biomarkers for the detection of severe forms of OSA.

The brain is one of the organs with a higher rate of oxygen consumption and is highly vulnerable to hypoxia. Acute hypoxia increases the formation of reactive oxygen species (ROS) in the brain [38]. This organ is mainly composed of lipids, and hypoxia-induced ROS may increase lipoxidation and interfere with the proper functioning of these biomolecules [39]. Lipoxidation is well documented in both the brain and blood of AD patients [19, 20]. In addition, the existence of a link between OSA and lipid dyshomeostasis has been supported by several observational and meta-analyses studies [18, 40].

Lipidomics has been used to study blood [41] and CSF [42] lipid alterations as biomarkers for AD diagnosis and differential diagnosis from other types of dementia. Metabolomics, another discipline that detects all types of metabolites, including lipids, has revealed that lipids are the most dysregulated class of metabolites in OSA [40]. Previous studies have detected phospholipids [43, 44], sphingolipids [45], and endocannabinoids [46] as biomarkers of OSA in cognitively unimpaired populations. These previous works have mainly used blood and urine samples for the detection of OSA biomarkers with the aim of detecting systemic lipid abnormalities caused by OSA. The search for OSA-related lipid alterations at the systemic level in patients with AD is scarce, and the only study conducted in this population showed lipid alterations in phospholipids and triglycerides with higher levels of oxidized lipid species in AD patients with severe OSA [47]. However, AD principally affects brain physiology and function. Therefore, when searching for OSA-associated lipid alterations in AD patients, CSF would be a valuable sample because of its proximity to the brain tissue, and probably any OSA-provoked lipid alteration specific to AD would be captured in CSF better than any other biological fluid. In addition, CSF is collected in clinical settings for the measurement of AD core biomarkers needed for the diagnosis of AD, and the same sample can be used for lipidomics without increasing the additional risk for the patients. As a result, we searched for a specific fingerprint of OSA in the CSF of AD patients. From 11 differentially expressed lipids between AD patients with and without severe OSA, we identified two: OxCer(40:6) and OxTG(57:2). These two lipid species were not related to either OSA or AD in previous studies. In addition, we did not find any information regarding the possible biological pathways that these two lipid species may be involved. The fact that both identified lipids were oxidized suggests that the increase in lipoxidation may be an important causative effect by which OSA exacerbates AD pathology or increases AD incidence, as reported previously [8, 48–50]. In addition, having high discriminating power in separating AD patients with different OSA severities highlights the promising future of these biomolecules as biomarkers of OSA.

Ceramides are structural constituents of the biological membranes and are also involved, as bioactive molecules, in a variety of biological events, including cell differentiation and proliferation, redox metabolism, inflammation, and apoptosis [51]. Ceramide regulates the BACE1-mediated processing of amyloid precursor protein (APP), probably by the formation of ceramide-enriched platforms and the enhancement of BACE1 stability in cells. Inhibition of sphingomyelinase (SMase), the enzyme that mediates the conversion of sphingomyelin (SM) to ceramide, inhibits γ-secretase activity and leads to a reduction in Aβ secretion [52, 53]. We found that higher levels of OxCer(40:6) in CSF were associated with the presence of severe OSA in AD patients. It has been suggested that the lipoxidation state is important for APP processing. When there is a high concentration of oxidized lipids, APP processing may shift from a nonamyloidogenic to an amyloidogenic pathway [52]. Therefore, severe forms of OSA, by inducing lipoxidation of membrane-associated lipids, such as Cer(40:6), may increase AD pathology. In addition, oxidation may alter other functions of ceramides related to inflammation, redox homeostasis, and cell death that may play a role in AD pathogenesis.

Our results also revealed lower levels of TGs in CSF, specifically OxTG(57:2), in AD patients with OSA severity. Importantly, there was not any significant difference in medication between the groups. Therefore, this observation may not be related to the medication use. Previous studies have reported significantly decreased serum TG levels in AD patients compared to a control group [54–56], which are, in addition, associated with early-AD biomarkers, including entorhinal cortical thickness and hippocampal volume measured by MRI scans, regions especially affected by AD. Our finding extends these previous observations from serum to CSF and suggests that impairments in TG metabolism among AD patients differ based on OSA severity. It remains unclear whether OSA severity also impairs the deterioration of the cortical parameters associated with AD. Inside cells, TGs are found in organelles called lipid droplets (LDs). In the brain, LDs have been found both in the neurons and in glial cells [57]. In the AD brain, it seems that the accumulation of LDs is more pronounced in the glia. Oxidative stress increases the number of LDs in glial cells, especially in astrocytes. Oxidized lipids produced in neurons are transported to surrounding astrocytes for detoxification and storage in LDs [58]. TGs stored in LDs can serve as a source of energy and cell signalling molecules [59]. In this context, it is proposed that the low TG levels in the CSF of AD patients with severe OSA express bioenergetic exhaustion of neural cells, with effects in oxidative stress conditions, which can favour cell death and the subsequent deterioration of brain functions and progression of AD. However, the specific mechanisms underlying the association of decreased TGs and AD and its impairment by OSA remain to be determined.

This untargeted approach is the initial step in this field of research and presents new knowledge from which new hypotheses can be generated. We believe that the validity of our findings should be determined by a targeted approach in an independent sample of AD subjects. We did not correct our data for multiple comparison, and from 11 differentially expressed lipids between the groups, we identified two lipid species. Future advances and accessibility to finer equipment will surely help to identify more lipid species related to OSA severity in AD. We included patients with MMSE scores > 20, so the results should be interpreted with caution when extrapolating to patients at more advanced stages of the disease. The main strength of this study was the use of PSG for the diagnosis of OSA in our population. This allowed us to perform correlation analysis to examine the association between different PSG variables and the severity of OSA. In addition, our study population was well-defined, and there was no significant difference regarding many sociodemographic characteristics and comorbidities that potentially would affect the results between the groups. Moreover, we included a relatively large sample of consecutive AD subjects, which increased the generalizability of the data.

## Conclusions

In this study, using undirected lipidomics, we identified a lipid profile in the CSF of severe OSA patients with mild-moderate AD that was correlated with different polysomnographic measures of OSA severity. From 11 differentially expressed lipids between the groups, we identified two oxidized lipid species. Therefore, our results suggest that an increase in lipoxidation may be one of the mechanisms by which OSA negatively affects AD physiopathology. Furthermore, we identified a lipidomic signature that allows the identification of AD patients with severe OSA in a population of AD with higher accuracy than the STOP-Bang screening scale. These data suggest that dysregulated lipid species in CSF could be potential biomarkers of OSA and could be used for screening those patients who could subsequently benefit from undergoing a PSG where there is a high suspicion of severe OSA.

### Supplementary Information

**Additional file 1: Table S1.** Class representative and extraction internal standards added to the samples. **Fig. S1.** Quality control of the included data. A) PCA of total lipid profile. B) Distribution of distance from centroid according to the groups. **Fig. S2.** Lipid features that significantly differentially expressed between AD patients with and without sever OSA. The unknown features are shown as mass at retention time. FC: fold change; OxCer: oxidized ceramide; OxTG: oxidized triglyceride. **Fig. S3.** Importance of each lipid in the classification of the study groups (severe OSA vs. nonsevere OSA) based on random forest. OxCer: oxidized ceramide; OxTG: oxidized triglyceride. **Fig. S4.** Correlations between levels of differentially expressed lipids in CSF and PSG parameters of OSA severity. The colour scale illustrates the degree of correlation and ranges from red to blue, indicating positive and negative correlations, respectively. Unknown features are presented as exact mass at retention time. AHI: apnoea-hypopnea index; BMI: body mass index; FC: fold change; OSA: obstructive sleep apnoea; OxTG: oxidized triglyceride; OxCer: oxidized ceramide; CT90: time with oxygen saturation < 90%. For interpretation of the references to colour in this figure legend, the reader is referred to the web version of this article. **Table S2.** Correlations between the levels of differentially expressed lipids in CSF and PSG parameters of OSA severity. **Fig. S5.** Lipid features most associated with the diagnosis of severe OSA based on multivariate regression analysis using a backward selection procedure.

## Data Availability

The data reported in this manuscript are available within the article and/or its supplementary data. Additional data from NCT02814045 will be shared by request from any qualified investigator.

## References

[1] Lane CA, Hardy J, Schott JM (2018). Alzheimer’s disease. Eur J Neurol.

[2] Nichols E, Steinmetz JD, Vollset SE, Fukutaki K, Chalek J, Abd-Allah F (2022). Estimation of the global prevalence of dementia in 2019 and forecasted prevalence in 2050: an analysis for the Global Burden of Disease Study 2019. Lancet Public Heal..

[3] Jack CR, Bennett DA, Blennow K, Carrillo MC, Dunn B, Haeberlein SB (2018). NIA-AA Research Framework: toward a biological definition of Alzheimer’s disease. Alzheimers Dement..

[4] Ferrer I. Hypothesis review: Alzheimer’s overture guidelines. Brain Pathol. 2022;e13122. 10.1111/bpa.13122 PMC9836379 36223647

[5] Edwards GA, Gamez N, Escobedo G, Calderon O, Moreno-Gonzalez I (2019). Modifiable risk factors for Alzheimer’s disease. Front Aging Neurosci.

[6] Gaeta AM, Benítez ID, Jorge C, Torres G, Dakterzada F, Minguez O (2020). Prevalence of obstructive sleep apnea in Alzheimer’s disease patients. J Neurol.

[7] Osorio RS, Ayappa I, Mantua J, Gumb T, Varga A, Mooney AM (2014). Interaction between sleep-disordered breathing and apolipoprotein E genotype on cerebrospinal fluid biomarkers for Alzheimer’s disease in cognitively normal elderly individuals. Neurobiol Aging..

[8] Diáz-Román M, Pulopulos MM, Baquero M, Salvador A, Cuevas A, Ferrer I (2021). Obstructive sleep apnea and Alzheimer’s disease-related cerebrospinal fluid biomarkers in mild cognitive impairment. Sleep..

[9] Sharma RA, Varga AW, Bubu OM, Pirraglia E, Kam K, Parekh A (2018). Obstructive sleep apnea severity affects amyloid burden in cognitively normal elderly: a longitudinal study. Am J Respir Crit Care Med..

[10] Osorio RS, Gumb T, Pirraglia E, Varga AW, Lu SE, Lim J (2015). Sleep-disordered breathing advances cognitive decline in the elderly. Neurology..

[11] Troussière AC, Charley CM, Salleron J, Richard F, Delbeuck X, Derambure P (2014). Treatment of sleep apnoea syndrome decreases cognitive decline in patients with Alzheimer’s disease. J Neurol Neurosurg Psychiatry..

[12] Jorge C, Targa A, Benítez ID, Dakterzada F, Torres G, Minguez O, et al. Obstructive sleep apnoea and cognitive decline in mild-to-moderate Alzheimer’s disease. Eur Respir J. 2020;56(5). 10.1183/13993003.00523-2020 32554539

[13] Gaines J, Vgontzas AN, Fernandez-Mendoza J, Bixler EO (2018). Obstructive sleep apnea and the metabolic syndrome: the road to clinically-meaningful phenotyping, improved prognosis, and personalized treatment. Sleep Med Rev..

[14] Loewen AHS, Korngut L, Rimmer K, Damji O, Turin TC, Hanly PJ (2014). Limitations of split-night polysomnography for the diagnosis of nocturnal hypoventilation and titration of non-invasive positive pressure ventilation in amyotrophic lateral sclerosis. Amyotroph Lateral Scler Frontotemporal Degener..

[15] Chung F, Yegneswaran B, Liao P, Chung SA, Vairavanathan S, Islam S (2008). STOP questionnaire: a tool to screen patients for obstructive sleep apnea. Anesthesiology..

[16] Netzer NC, Stoohs RA, Netzer CM, Clark K, Strohl KP (1999). Using the Berlin Questionnaire to identify patients at risk for the sleep apnea syndrome. Ann Intern Med..

[17] Jorge C, Benítez ID, Torres G, Dakterzada F, Minguez O, Huerto R (2019). The STOP-Bang and Berlin questionnaires to identify obstructive sleep apnoea in Alzheimer’s disease patients. Sleep Med..

[18] Barros D, García-Río F (2019). Obstructive sleep apnea and dyslipidemia: from animal models to clinical evidence. Sleep..

[19] Zabel M, Nackenoff A, Kirsch WM, Harrison FE, Perry G, Schrag M (2018). Markers of oxidative damage to lipids, nucleic acids and proteins and antioxidant enzymes activities in Alzheimer’s disease brain: a meta-analysis in human pathological specimens. Free Radic Biol Med..

[20] Schrag M, Mueller C, Zabel M, Crofton A, Kirsch WM, Ghribi O (2013). Oxidative stress in blood in Alzheimer’s disease and mild cognitive impairment: a meta-analysis. Neurobiol Dis..

[21] McKhann GM, Knopman DS, Chertkow H, Hyman BT, Jack CR, Kawas CH (2011). The diagnosis of dementia due to Alzheimer’s disease: recommendations from the National Institute on Aging-Alzheimer’s Association workgroups on diagnostic guidelines for Alzheimer’s disease. Alzheimers Dement..

[22] Folstein MF, Folstein SE, McHugh PR (1975). “Mini-mental state”: a practical method for grading the cognitive state of patients for the clinician. J Psychiatr Res.

[23] Johns MW (1991). A new method for measuring daytime sleepiness: the Epworth Sleepiness Scale. Sleep..

[24] Ortega RL, Dakterzada F, Arias A, Blasco E, Naudí A, Garcia FP (2019). Usefulness of CSF biomarkers in predicting the progression of amnesic and nonamnesic mild cognitive impairment to Alzheimer’s disease. Curr Aging Sci.

[25] Pizarro C, Arenzana-Rámila I, Pérez-Del-Notario N, Pérez-Matute P, González-Sáiz JM (2013). Plasma lipidomic profiling method based on ultrasound extraction and liquid chromatography mass spectrometry. Anal Chem..

[26] Sol J, Jové M, Povedano M, Sproviero W, Domínguez R, Piñol-Ripoll G, et al. Lipidomic traits of plasma and cerebrospinal fluid in amyotrophic lateral sclerosis correlate with disease progression. Brain Commun. 2021;3(3). 10.1093/braincomms/fcab143 PMC8361390 34396104

[27] Castro-Perez JM, Kamphorst J, Degroot J, Lafeber F, Goshawk J, Yu K (2010). Comprehensive LC-MS E lipidomic analysis using a shotgun approach and its application to biomarker detection and identification in osteoarthritis patients. J Proteome Res..

[28] Sandra K, Pereira A dos S, Vanhoenacker G, David F, Sandra P (2010). Comprehensive blood plasma lipidomics by liquid chromatography/quadrupole time-of-flight mass spectrometry. J Chromatogr A..

[29] Jové M, Cabré R, Mota-Martorell N, Martin-Garí M, Obis È, Ramos P (2021). Age-related changes in lipidome of rat frontal cortex and cerebellum are partially reversed by methionine restriction applied in old age. Int J Mol Sci..

[30] Broadhurst D, Goodacre R, Reinke SN, Kuligowski J, Wilson ID, Lewis MR, et al. Guidelines and considerations for the use of system suitability and quality control samples in mass spectrometry assays applied in untargeted clinical metabolomic studies. Metabolomics. 2018;14(6). 10.1007/s11306-018-1367-3 PMC5960010 29805336

[31] Dunn WB, Broadhurst D, Begley P, Zelena E, Francis-Mcintyre S, Anderson N (2011). Procedures for large-scale metabolic profiling of serum and plasma using gas chromatography and liquid chromatography coupled to mass spectrometry. Nat Protoc.

[32] Wishart DS, Feunang YD, Marcu A, Guo AC, Liang K, Vázquez-Fresno R (2018). HMDB 4.0: the human metabolome database for 2018. Nucleic Acids Res..

[33] Ritchie ME, Phipson B, Wu D, Hu Y, Law CW, Shi W (2015). limma powers differential expression analyses for RNA-sequencing and microarray studies. Nucleic Acids Res..

[34] Genuer R, Poggi JM, Tuleau-Malot C (2010). Variable selection using random forests. Pattern Recognit Lett.

[35] Bubu OM, Andrade AG, Umasabor-Bubu OQ, Hogan MM, Turner AD, de Leon MJ (2020). Obstructive sleep apnea, cognition and Alzheimer’s disease: a systematic review integrating three decades of multidisciplinary research. Sleep Med Rev..

[36] Redline S (2017). Screening for obstructive sleep apnea: implications for the sleep health of the population. JAMA..

[37] Arnaud C, Bochaton T, Pépin JL, Belaidi E (2020). Obstructive sleep apnoea and cardiovascular consequences: pathophysiological mechanisms. Arch Cardiovasc Dis..

[38] Coimbra-Costa D, Alva N, Duran M, Carbonell T, Rama R (2017). Oxidative stress and apoptosis after acute respiratory hypoxia and reoxygenation in rat brain. Redox Biol..

[39] Lavie L (2015). Oxidative stress in obstructive sleep apnea and intermittent hypoxia–revisited–the bad ugly and good: implications to the heart and brain. Sleep Med Rev..

[40] Humer E, Pieh C, Brandmayr G (2020). Metabolomics in sleep, insomnia and sleep apnea. Int J Mol Sci..

[41] Liu Y, Thalamuthu A, Mather KA, Crawford J, Ulanova M, Wong MWK (2021). Plasma lipidome is dysregulated in Alzheimer’s disease and is associated with disease risk genes. Transl Psychiatry.

[42] Byeon SK, Madugundu AK, Jain AP, Bhat FA, Jung JH, Renuse S (2021). Cerebrospinal fluid lipidomics for biomarkers of Alzheimer’s disease. Mol Omi..

[43] Ferrarini A, Rupérez FJ, Erazo M, Martínez MP, Villar-Álvarez F, Peces-Barba G (2013). Fingerprinting-based metabolomic approach with LC-MS to sleep apnea and hypopnea syndrome: a pilot study. Electrophoresis..

[44] Pinilla L, Benítez ID, Santamaria-Martos F, Targa A, Moncusí-Moix A, Dalmases M, et al. Plasma profiling reveals a blood-based metabolic fingerprint of obstructive sleep apnea. Biomed Pharmacother. 2022;145. 10.1016/j.biopha.2021.112425 34800782

[45] Lebkuchen A, Carvalho VM, Venturini G, Salgueiro JS, Freitas LS, Dellavance A (2018). Metabolomic and lipidomic profile in men with obstructive sleep apnoea: implications for diagnosis and biomarkers of cardiovascular risk. Sci Rep..

[46] Engeli S, Blüher M, Jumpertz R, Wiesner T, Wirtz H, Bosse-Henck A (2012). Circulating anandamide and blood pressure in patients with obstructive sleep apnea. J Hypertens..

[47] Dakterzada F, Benítez ID, Targa A, Carnes A, Pujol M, Jové M, et al. Blood-based lipidomic signature of severe obstructive sleep apnoea in Alzheimer’s disease. Alzheimers Res Ther. 2022;14(1):163. 10.1186/s13195-022-01102-8 PMC9632042 36329512

[48] Bubu OM, Pirraglia E, Andrade AG, Sharma RA, Gimenez-Badia S, Umasabor-Bubu OQ (2019). Obstructive sleep apnea and longitudinal Alzheimer’s disease biomarker changes. Sleep..

[49] Yaffe K, Laffan AM, Harrison SL, Redline S, Spira AP, Ensrud KE (2011). Sleep-disordered breathing, hypoxia, and risk of mild cognitive impairment and dementia in older women. JAMA - J Am Med Assoc.

[50] Emamian F, Khazaie H, Tahmasian M, Leschziner GD, Morrell MJ, Hsiung GYR, et al. The association between obstructive sleep apnea and Alzheimer’s disease: a meta-analysis perspective. Front Aging Neurosci. 2016;8(APR):78. 10.3389/fnagi.2016.00078 PMC4828426 27148046

[51] Stiban J (2019). Introduction: enigmas of sphingolipids. Adv Exp Med Biol..

[52] Chew H, Solomon VA, Fonteh AN (2020). Involvement of lipids in Alzheimer’s disease pathology and potential therapies. Front Physiol.

[53] Di Paolo G, Kim TW (2011). Linking lipids to Alzheimer’s disease: cholesterol and beyond. Nat Rev Neurosci..

[54] Hall K, Murrell J, Ogunniyi A, Deeg M, Baiyewu O, Gao S (2006). Cholesterol, APOE genotype, and Alzheimer disease: an epidemiologic study of Nigerian Yoruba. Neurology..

[55] Lepara O, Valjevac A, Alajbegović A, Zaćiragic A, Nakaś-Ićindic E (2009). Decreased serum lipids in patients with probable Alzheimer’s disease. Bosn J basic Med Sci..

[56] Bernath MM, Bhattacharyya S, Nho K, Barupal DK, Fiehn O, Baillie R (2020). Serum triglycerides in Alzheimer disease: relation to neuroimaging and CSF biomarkers. Neurology..

[57] Farmer BC, Walsh AE, Kluemper JC, Johnson LA. Lipid droplets in neurodegenerative disorders. Front Neurosci. 2020;14. 10.3389/fnins.2020.00742 PMC7403481 32848541

[58] Ioannou MS, Jackson J, Sheu SH, Chang CL, Weigel AV, Liu H (2019). Neuron-astrocyte metabolic coupling protects against activity-induced fatty acid toxicity. Cell..

[59] Olzmann JA, Carvalho P (2018). Dynamics and functions of lipid droplets. Nat Rev Mol Cell Biol.

