# Supplementary material for: Cerebrospinal fluid lipidomic fingerprint of obstructive sleep apnoea in Alzheimer’s disease
Source: Alzheimers Res Ther. 2023 Aug 7;15:134. doi: 10.1186/s13195-023-01278-7 (PMC10408111; doi:10.1186/s13195-023-01278-7)
Supplement: Supplementary file 1 — Additional file 1: Table S1. Class representative and extraction internal standards added to the samples. Fig. S1. Quality control of the included data. A) PCA of total lipid profile. B) Distribution of distance from centroid according to the groups. Fig. S2. Lipid features that significantly differentially expressed between AD patients with and without sever OSA. The unknown features are shown as mass at retention time. FC: fold change; OxCer: oxidized ceramide; OxTG: oxidized triglyceride. Fig. S3. Importance of each lipid in the classification of the study groups (severe OSA vs. nonsevere OSA) based on random forest. OxCer: oxidized ceramide; OxTG: oxidized triglyceride. Fig. S4. Correlations between levels of differentially expressed lipids in CSF and PSG parameters of OSA severity. The colour scale illustrates the degree of correlation and ranges from red to blue, indicating positive and negative correlations, respectively. Unknown features are presented as exact mass at retention time. AHI: apnoea-hypopnea index; BMI: body mass index; FC: fold change; OSA: obstructive sleep apnoea; OxTG: oxidized triglyceride; OxCer: oxidized ceramide; CT90: time with oxygen saturation < 90%. For interpretation of the references to colour in this figure legend, the reader is referred to the web version of this article. Table S2. Correlations between the levels of differentially expressed lipids in CSF and PSG parameters of OSA severity. Fig. S5. Lipid features most associated with the diagnosis of severe OSA based on multivariate regression analysis using a backward selection procedure. [file 13195_2023_1278_MOESM1_ESM.docx]

**Cerebrospinal Fluid Lipidomic Fingerprint of Obstructive Sleep Apnoea in Alzheimer’s Disease**

Farida Dakterzada^1^, Iván D. Benítez^2,3^, Adriano Targa^2,3^, Anna Carnes^1^, Montse Pujol^2^, Mariona Jové^4^, Olga Mínguez^2^, Rafi Vaca^2^, Manuel Sánchez‑de‑la‑Torre^5^, Ferran Barbé^2,3^, Reinald Pamplona^4^ and Gerard Piñol‑Ripoll^1^*

1. Unitat Trastorns Cognitius, Cognition and Behaviour Study Group, Santa Maria University Hospital, IRBLleida, Rovira Roure No. 44, 25198, Lleida, Spain
2. Group of Translational Research in Respiratory Medicine, Hospital Universitari Arnau de Vilanova and Santa Maria, IRBLleida, Lleida, Spain
3. Center for Biomedical Research in Respiratory Diseases Network (CIBERES), Madrid, Spain
4. Department of Experimental Medicine, University of Lleida-Biomedical Research Institute of Lleida (UdL-IRBLleida), Lleida, Spain
5. Department of Nursing and Physiotherapy, Group of Precision Medicine in Chronic Diseases, University Hospital Arnau de Vilanova and Santa María, IRBLleida, Faculty of Nursing and Physiotherapy, University of Lleida, Lleida, Spain

* Corresponding author:

Gerard Piñol-Ripoll

Cognitive Disorders Unit

Hospital Universitari Santa Maria.

Rovira Roure n° 44. 25198. Lleida. Spain

Telephone: 34-937-727222. Ext. 173. Fax: 34-976-727366

E-mail: gerard_437302@hotmail.com

**Supplementary Table 1.** Class representative and extraction internal standards added to the samples.

| **Compound** | **Reference**  **(Catalogue number, provider)** |
| --- | --- |
| 1,3(d5)-dihexadecanoyl-glycerol | 110537, Avanti Polar Lipids |
| 1,3(d5)-dihexadecanoyl-2-octadecanoyl-glycerol | 110543, Avanti Polar Lipids |
| 1-hexadecanoyl(d31)-2-(9Z-octadecenoyl)-sn-glycero-3-phosphate | 110920, Avanti Polar Lipids |
| 1-hexadecanoyl(d31)-2-(9Z-octadecenoyl)-sn-glycero-3-phosphocholine | 110918, Avanti Polar Lipids |
| 1-hexadecanoyl(d31)-2-(9Z-octadecenoyl)-sn-glycero-3-phosphoethanolamine | 110921, Avanti Polar Lipids |
| 1-hexadecanoyl-2-(9Z-octadecenoyl)-sn-glycero-3-phospho-(1'-rac-glycerol-1',1',2',3',3'-d5) | 110899, Avanti Polar Lipids |
| 1-hexadecanoyl(d31)-2-(9Z-octadecenoyl)-sn-glycero-3-phospho-myo-inositol | 110923, Avanti Polar Lipids |
| 1-hexadecanoyl(d31)-2-(9Z-octadecenoyl)-sn-glycero-3-[phospho-L-serine] | 110922, Avanti Polar Lipids |
| 26:0-d4 Lyso PC | 860389, Avanti Polar Lipids |
| 18:1 Chol (D7) ester | 111015, Avanti Polar Lipids |
| cholest-5-en-3ß-ol(d7) | LM-4100, Avanti Polar Lipids |
| D-erythro-sphingosine-d7 | 860657, Avanti Polar Lipids |
| D-erythro-sphingosine-d7-1-phosphate | 860659, Avanti Polar Lipids |
| N-palmitoyl-d31-D-erythro-sphingosine | 868516, Avanti Polar Lipids |
| N-palmitoyl-d31-D-erythro-sphingosylphosphorylcholine | 868584, Avanti Polar Lipids |
| Octadecanoic acid-2,2-d2 | 19905-58-9, Sigma Aldrich |


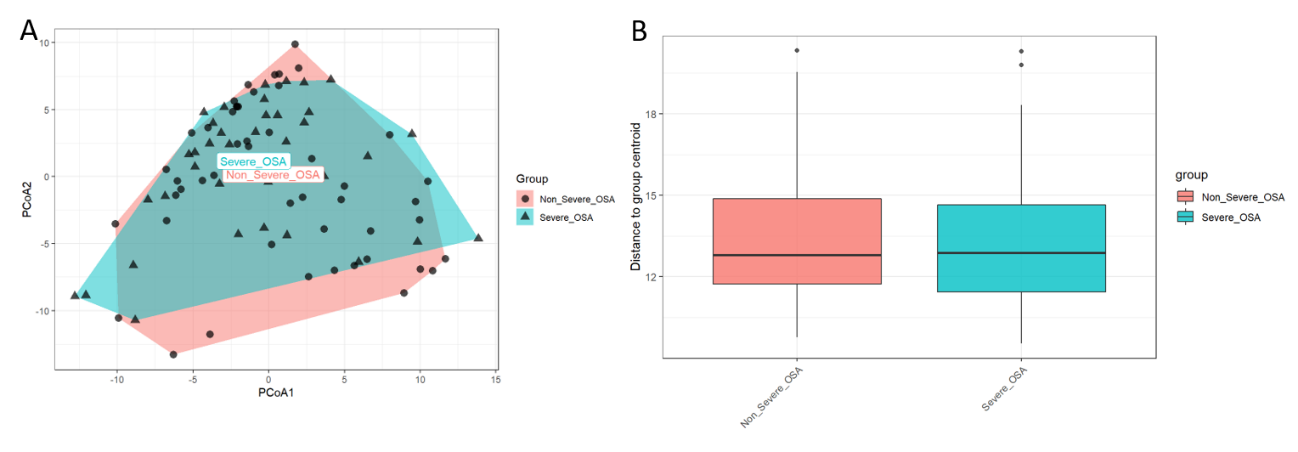


**Supplementary Figure 1.** Quality control of the included data. A) PCA of total lipid profile. B) Distribution of distance from centroid according to the groups.

**Supplementary Figure 2.** Lipid features that significantly differentially expressed between AD patients with and without sever OSA. The unknown features are shown as mass at retention time. FC: fold change; OxCer: oxidized ceramide; OxTG: oxidized triglyceride


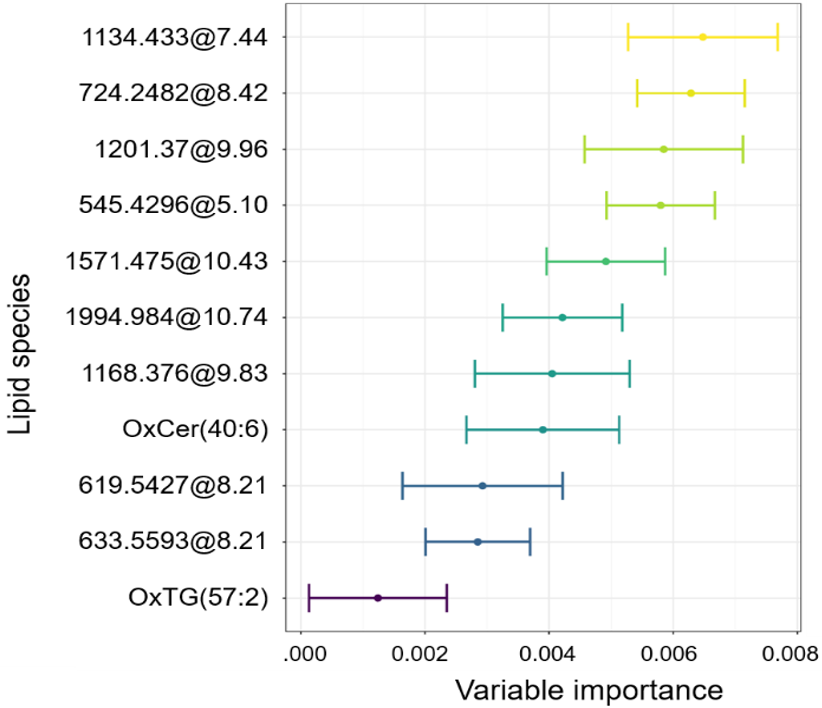


**Supplementary Figure 3.** Importance of each lipid in the classification of the study groups (severe OSA vs. nonsevere OSA) based on random forest. OxCer: oxidized ceramide; OxTG: oxidized triglyceride.


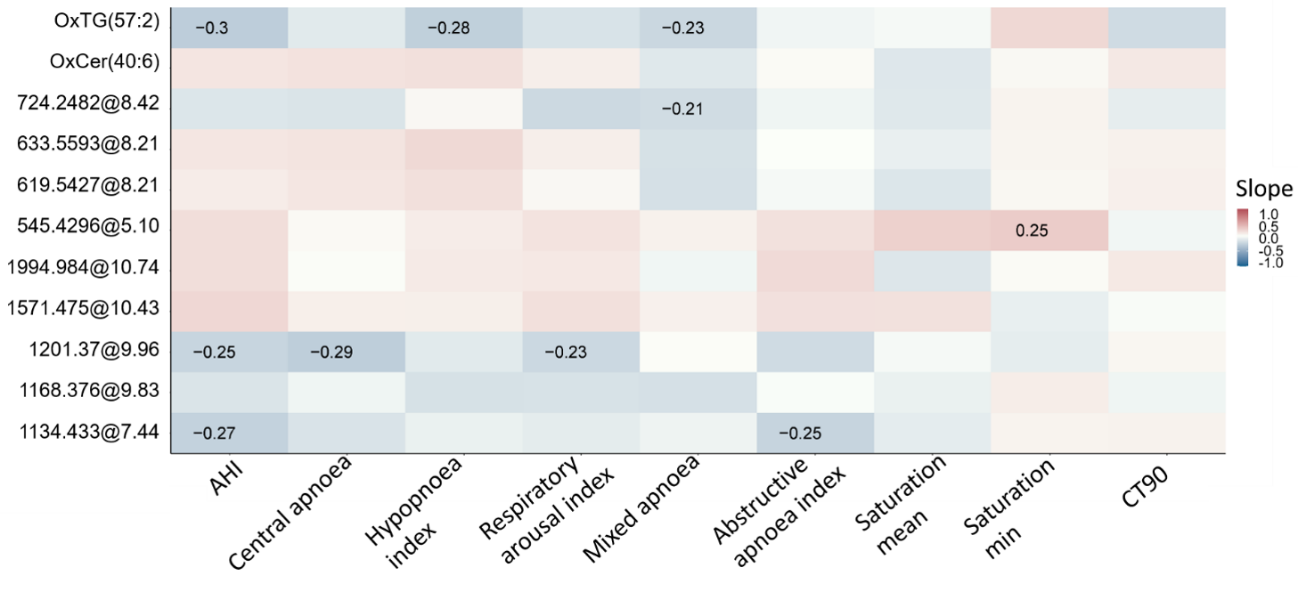


**Supplementary Figure 4.** Correlations between levels of differentially expressed lipids in CSF and PSG parameters of OSA severity. The colour scale illustrates the degree of correlation and ranges from red to blue, indicating positive and negative correlations, respectively. Unknown features are presented as exact mass at retention time. AHI: apnoea-hypopnea index; BMI: body mass index; FC: fold change; OSA: obstructive sleep apnoea; OxTG: oxidized triglyceride; OxCer: oxidized ceramide; CT90: time with oxygen saturation <90%. For interpretation of the references to colour in this figure legend, the reader is referred to the web version of this article.

**Supplementary Table 2.** Correlations between the levels of differentially expressed lipids in CSF and PSG parameters of OSA severity.

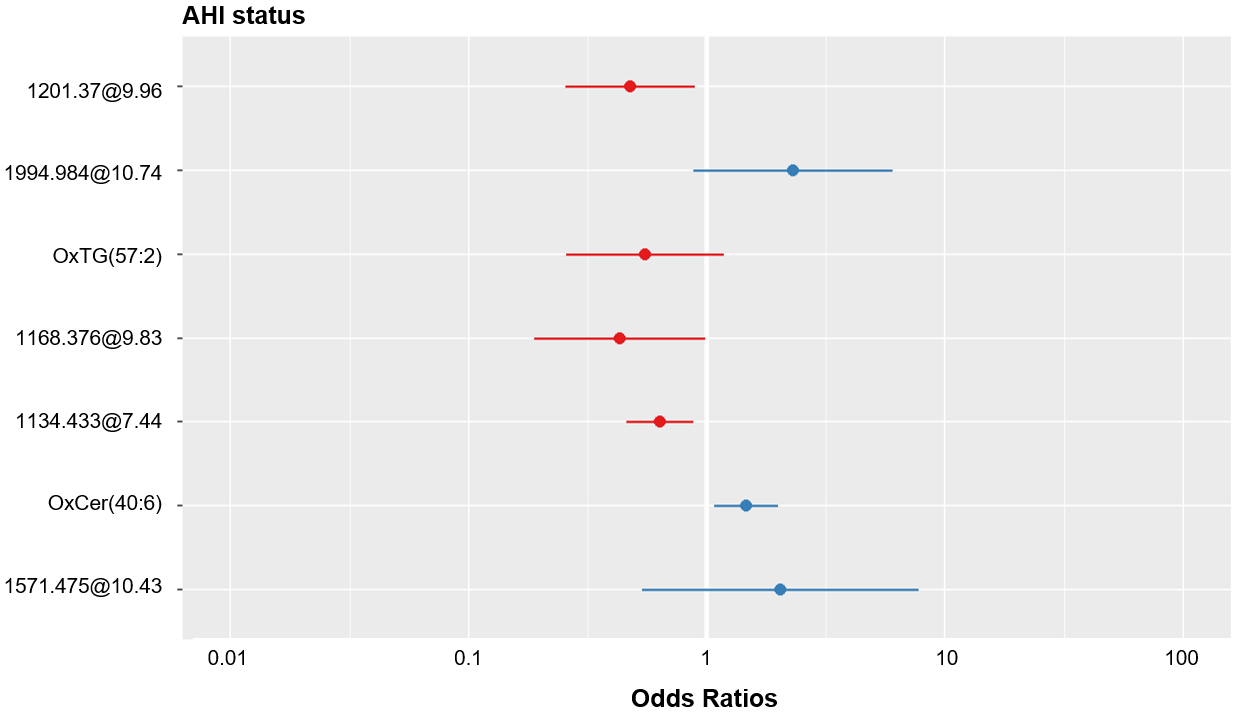


**Supplememntary Figure 5.** Lipid features most associated with the diagnosis of severe OSA based on multivariate regression analysis using a backward selection procedure.
